# Research on Acid Aging and Damage Pattern Recognition of Glass Fiber-Reinforced Plastic Oil and Gas Gathering Pipelines Based on Acoustic Emission

**DOI:** 10.3390/polym16162272

**Published:** 2024-08-10

**Authors:** Haisheng Bi, Yuhong Zhang, Chen Zhang, Chunxun Ma, Yuxiang Li, Jiaxu Miao, Guang Wang, Haoran Cheng

**Affiliations:** 1 College of Electromechanical Engineering, Qingdao University of Science and Technology, Qingdao 266061, China; zyh3451086638@163.com (Y.Z.); zhangchenczc@163.com (C.Z.); 13276396281@163.com (C.M.); q1425871511@163.com (Y.L.); 18265968386@163.com (G.W.); 2 College of Pipeline and Civil Engineering, China University of Petroleum (Huadong), Qingdao 266580, China; mjx040017@163.com; 3 Sichuan Energy Internet Research Institute, Tsinghua University, Chengdu 610218, China; 4 School of Chemical Engineering, Qingdao University of Science and Technology, Qingdao 266042, China

**Keywords:** GFRP pipe, pattern recognition, four-point bend, acoustic emission, waveform analysis

## Abstract

Pipelines extend thousands of kilometers to transport and distribute oil and gas. Given the challenges often faced with corrosion, fatigue, and other issues in steel pipes, the demand for glass fiber-reinforced plastic (GFRP) pipes is increasing in oil and gas gathering and transmission systems. However, the medium that is transported through these pipelines contains multiple acid gases such as CO_2_ and H_2_S, as well as ions including Cl^−^, Ca^2+^, Mg^2+^, SO_4_^2−^, CO_3_^2−^, and HCO_3_^−^. These substances can cause a series of problems, such as aging, debonding, delamination, and fracture. In this study, a series of aging damage experiments were conducted on V-shaped defect GFRP pipes with depths of 2 mm and 5 mm. The aging and failure of GFRP were studied under the combined effects of external force and acidic solution using acoustic emission (AE) techniques. It was found that the acidic aging solution promoted matrix damage, fiber/matrix desorption, and delamination damage in GFRP pipes over a short period. However, the overall aging effect was relatively weak. Based on the experimental data, the SSA-LSSVM algorithm was proposed and applied to the damage pattern recognition of GFRP. An average recognition rate of up to 90% was achieved, indicating that this method is highly suitable for analyzing AE signals related to GFRP damage.

## 1. Introduction

With the persistent challenges of corrosion, fatigue, and other issues in steel pipes, the demand for glass fiber-reinforced plastic (GFRP) pipes has increased in oil and gas gathering and transmission systems [1,2,3]. However, under the conditions of pressure loading and aging of the transmission medium, GFRP pipelines also experience damage and failure issues, such as delamination and matrix cracking [4,5,6], which can easily lead to pipeline safety accidents.

Numerous scholars have studied the mechanical properties of GFRP pipes during long-term aging [7,8,9]. Solis et al. [10] designed a stress corrosion cracking (SCC) four-point bend self-loaded fixture to study the performance of aged glass-reinforced polymer composite materials and found that the residual bending strength and rigidity of the materials had been reduced. However, there is limited research on damage to GFRP materials under simultaneous external load and aging conditions. André et al. [11] used acoustic emission technology to reveal different corrosion modes of glass fibers in HCl solution, demonstrating the high efficiency of AE technology for the active corrosion detection of glass fibers.

In recent years, clustering analysis methods have been increasingly used in the classification of acoustic emission (AE) data. Numerous international experts and scholars have conducted extensive studies on the damage mechanisms and failure modes of GFRP composite materials using AE technology [12,13,14]. They have determined that parameters such as amplitude, ringing count, energy, and duration effectively reflect material damage. The K-means clustering algorithm is commonly applied to classify AE signals. For instance, Seif [15] extracted the frequency characteristics of three-point bending damage signals from unidirectional glass fiber-reinforced polymer composite samples using the Hilbert–Huang Transform (HHT). The author classified matrix cracking, fiber/matrix debonding, fiber fracture, and delamination signals based on the K-means clustering algorithm, demonstrating the robustness of AE data classification using the HHT. Pashmforoush et al. [16] combined the K-means algorithm with a genetic algorithm to cluster AE signals of I-type delamination damage in sandwich composite materials. They verified the effectiveness of this clustering method using scanning electron microscopy and identified four frequency bands (30–80 kHz, 100–150 kHz, 160–250 kHz, and 250–500 kHz) corresponding to core material cracking, panel–core material debonding, matrix cracking, and fiber fracture damage. Momon et al. [17] used a clustering analysis to derive the damage modes of C/SiC composites at high temperatures. Monti et al. [18] investigated the mechanical properties of composites composed of glass fiber-reinforced thermoplastic matrices under uniaxial tensile loading using AE techniques. They post-processed AE signals using a K-means clustering recognition algorithm to clarify the correlation between AE event categories and observed damage mechanisms.

At present, AE technology is widely used in the petrochemical, aerospace, transportation, construction, and power industries, among other fields. It involves the detection of metal materials, non-metallic materials, and composite materials such as GFRP [19,20,21,22,23,24,25]. Research on GFRP primarily focuses on aging experiments based on morphological analysis and stress damage experiments using acoustic emission. However, there are relatively few studies investigating the damage and aging processes of glass fiber materials under the coupling of aging and stress using AE technology.

In this study, GFRP pipes were used as experimental materials, and a four-point bending experimental device was designed. AE technology was employed to detect the real-time damage of GFRP under aging solution and external load. The K-means clustering algorithm was utilized to accurately classify the damage and aging signals of GFRP materials. This allowed for the analysis and study of the characteristics of AE signals and types of material damage. Additionally, the SSA-LSSVM algorithm was applied to identify the damage modes of GFRP materials. Taking the aging failure of GFRP pipes for gathering and transmission as the background, the aging effect of corrosive ions on the specimen of GFRP pipes for gathering and transmission systems under stress was studied by means of acoustic emission technology. The aging test duration of each specimen under the stress loading condition was 78 h. In the future, the field gathering and transmission medium can be used as the aging solution to monitor the specimens for a longer time and further improve the pipeline aging database for the identification of the damage degree and damage type of GFRP pipes.

## 2. Materials and Methods 

### 2.1. Experimental Materials and Treatment

**Figure 1:**
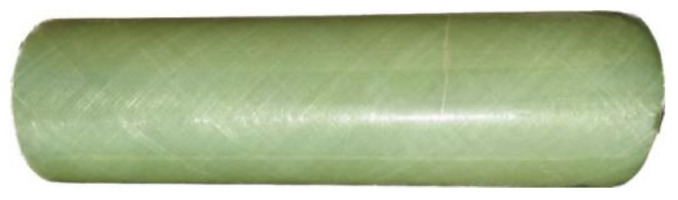
Experimental materials.

In this experiment, GFRP pipes used in the oilfield are selected as the experimental materials. Using epoxy resin as the matrix and glass fiber as the reinforcement, the pipe has a series of advantages, such as strong corrosion resistance, small pipe resistance, and light weight. As shown in Figure 1, the GFRP pipe is divided into three main layers: the inner layer, the inner winding layer, and the outer winding layer. The inner winding layer consists of three sub-layers, each with a thickness of 1 mm. The specific parameters of the GFRP pipe are detailed in Table 1.

**Figure 2:**
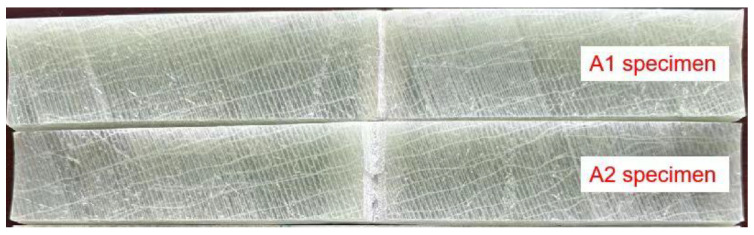
GFRP specimens.

**Figure 3:**
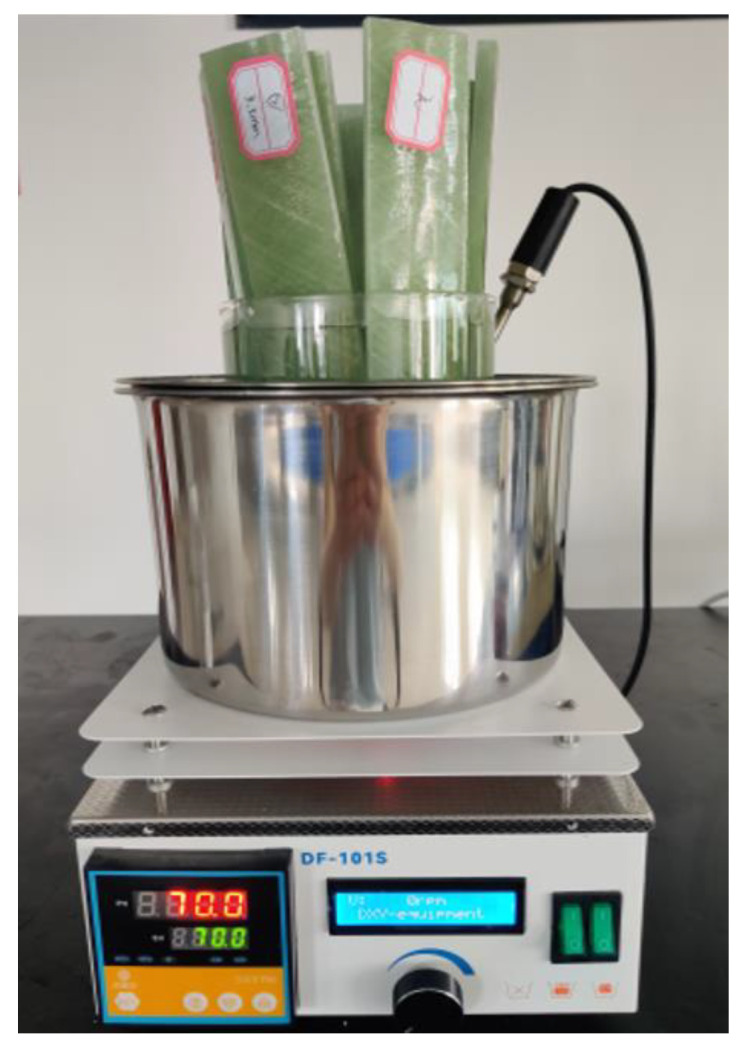
The aging device of GFRP specimens.

GFRP pipes were cut into 250 × 40 × 7.5 mm specimens using a CNC machine. V-shaped defects with an angle of 60° at the center of the specimens and depths of 2 mm and 5 mm were prefabricated. Figure 2 shows the V-shaped defect specimens. The processed specimens were placed in an aging device for 10 days. In order to isolate the aging solution, one end (isolation zone) of the specimen was wrapped with tin foil and fixed. The specific procedure is as follow. Firstly, an appropriate amount of purified water is poured into a magnetic stirrer, and then the beaker containing the aging solution is placed into the water. Secondly, the GFRP specimens were vertically immersed in the aging solution, ensuring that both the isolation zone and the aging zone of the specimens were in contact with the aging solution. Finally, the specimens were subjected to a 10-day aging pretreatment using an aging solution with a temperature of 70 °C and a pH value of 5.0. Figure 3 shows the aging apparatus used for GFRP specimens.

### 2.2. Experimental Aging Solution

Through investigating the components of field-produced fluids and the water formation in oilfields, it has been observed that the medium is predominantly acidic and contains Cl^−^, HCO_3_^−^, Na^+^, Ca^2+^, and Mg^2+^. Therefore, for this experiment, 1 L of aging solution was prepared using 23.4 g of NaCl, 1.092 g of NaHCO_3_, 1.221 g of CaCl_2_, and 8.118 g of MgSO_4_ 7H_2_O, and the pH value of the aging solution was adjusted to 5.0 using CH_3_COOH. The specific composition of the prepared aging solution is detailed in Table 2.

### 2.3. Experimental Device

**Figure 4:**
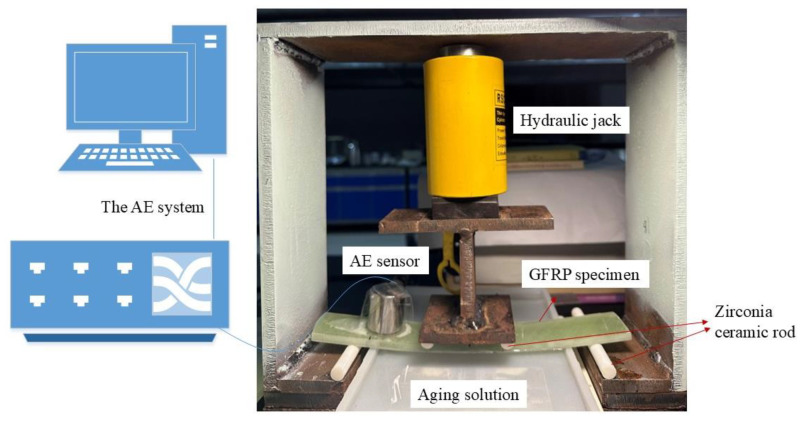
Loading diagram of the experimental device.

Based on the Standard Test Method for Flexural Properties of Polymer Matrix Composite Materials, a four-point loading configuration was utilized as depicted in Figure 4. In this configuration, the GFRP specimen is supported at two points and loaded at two points, each equidistant from the adjacent support point. The load span is half the distance between the support points.

During the experiment, the GFRP specimen was positioned such that the middle defect at the bottom was submerged in the aging solution. An AE (acoustic emission) sensor was securely affixed to the upper surface of the specimen and connected to the AE system (PAC, USA). The experiment involved measuring and recording the force applied to the GFRP specimen and the resulting deflection at the center of the span until failure occured on either outer surface.

This setup allows for the study of the damage characteristics and aging mechanism of GFRP under acidic aging conditions and a specific loading configuration using AE technology.

### 2.4. Experimental Process

This study utilized a four-point bending device to apply stress to pre-damaged specimens with a 2 mm depth V-shaped defect until they reached a significant matrix cracking state. Stress was applied to pre-damaged GFRP specimens with a 5 mm depth V-shaped defect until they exhibited a macroscopic delamination state. A 72 h acid aging experiment was conducted on the damaged part of the specimens, and the acoustic emission signal characteristics of the specimens’ damage and aging process were collected in real time using an acoustic emission instrument. The specific specimen design is shown in Table 3.

## 3. Results and Discussion

### 3.1. Microscopic Morphology Analysis

**Figure 5:**
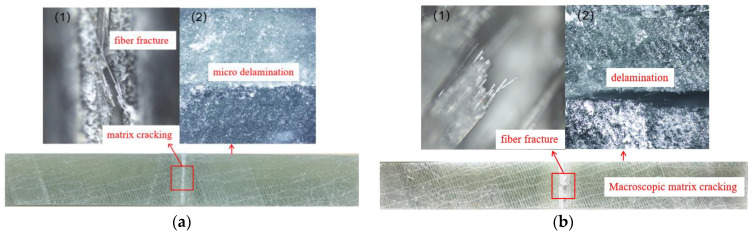
Microscopic observation of specimen. (**a**) A1 specimen; (**b**) A2 specimen.

The loading stress primarily induces visible damage morphology on the specimen after aging, while the aging solution does not visibly alter the material’s surface morphology over a short period. As depicted in Figure 5, the post-experiment damage morphology of the specimen reveals two perspectives: (1) surface damage morphology and (2) side damage morphology. For A1 specimens, surface observations reveal matrix cracking and fiber fracture damage, with slight delamination damage being visible on the side. However, A2 specimens exhibit more severe damage. Clear matrix cracks and fiber fractures are prominently visible at the central V-shaped pretreated area, accompanied by distinct delamination characteristics along the specimen’s side.

### 3.2. AE Parameter Analysis

Before the acid aging test, the specimen underwent loading to various degrees of damage, during which acoustic emission (AE) signals were collected. It was observed that the AE hits decreased significantly after approximately 18 h. Subsequently, the collected AE signals predominantly reflected the aging effects of the acid solution. Therefore, the entire procedure was divided into two stages: the stress damage stage and the aging stage. Signals collected from stage 2 to stage 5 were categorized as aging AE signals.

**Figure 6:**
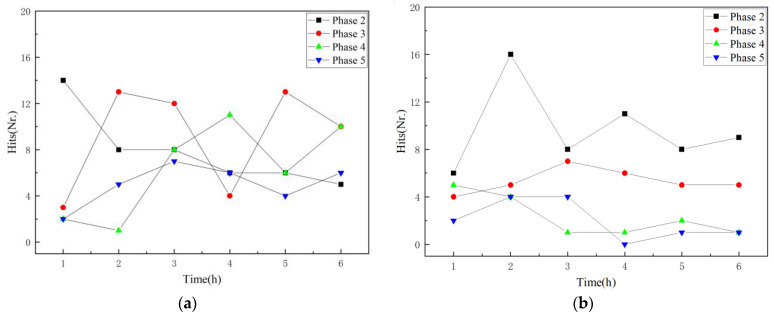
Hit distribution of the aging signal for the GFRP pipe. (**a**) A1 specimen; (**b**) A2 specimen.

Figure 6 illustrates the AE hits of the GFRP specimens at different aging stages over time. It is evident that there is no significant variation in the number of AE hits for GFRP specimens across the four stages. The AE hits consistently remain at a relatively low level, consistently remaining below 20. This observation reaffirms the accelerating effect of the acidic solution during the aging stage.

**Figure 7:**
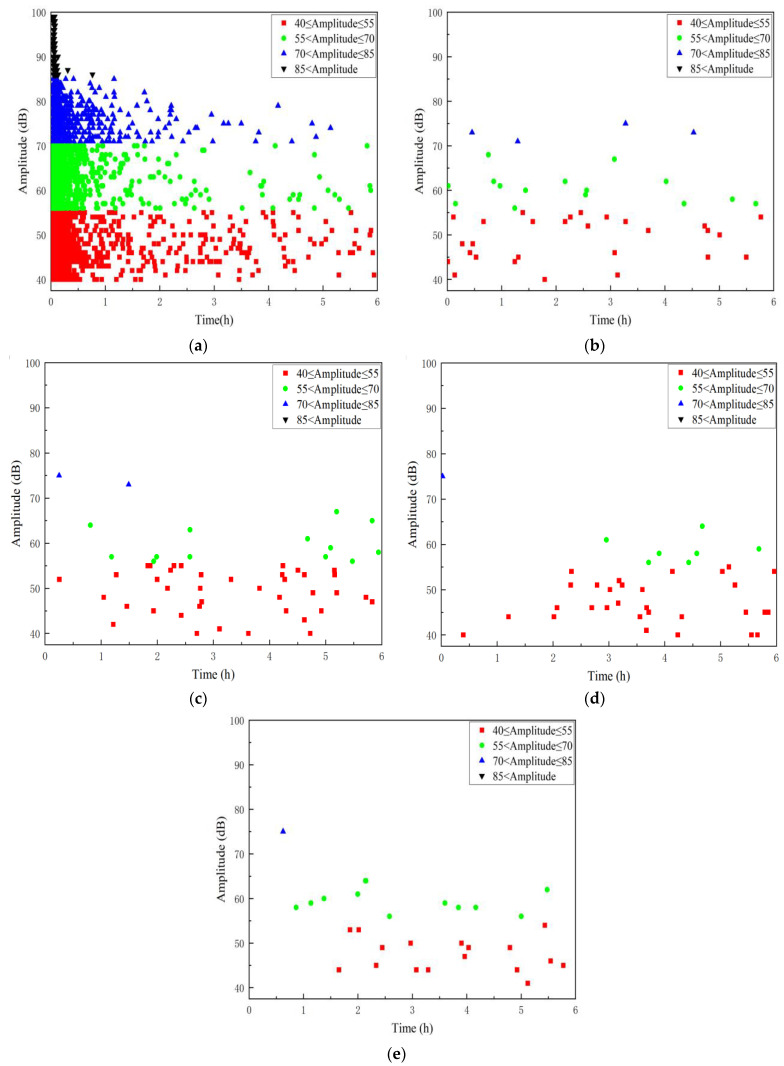
Time–Amplitude distribution of the damage AE signal in A1 specimen. (**a**) Phase 1; (**b**) Phase 2; (**c**) Phase 3; (**d**) Phase 4; (**e**) Phase 5.

In Figure 7, the time–amplitude relationship of the AE signal reveals the progression of specimen damage during the initial loading stage, where high signal amplitudes peak at 99 dB. During the first stage, AE signals indicating GFRP damage gradually diminish as mechanical stress-induced damage subsides within an hour. Concurrently, the aging effect of the acid solution on the GFRP specimen begins to manifest, characterized by AE signal amplitudes mainly ranging from 40 to 80 dB. Subsequently, AE signals during the aging stages (from stage 2 to stage 5) stabilize, with amplitudes consistently falling within the range of 40 to 70 dB. This pattern suggests a relatively stable state of aging-induced AE signals in the materials as the acid solution continues to exert its effects over time.

**Figure 8:**
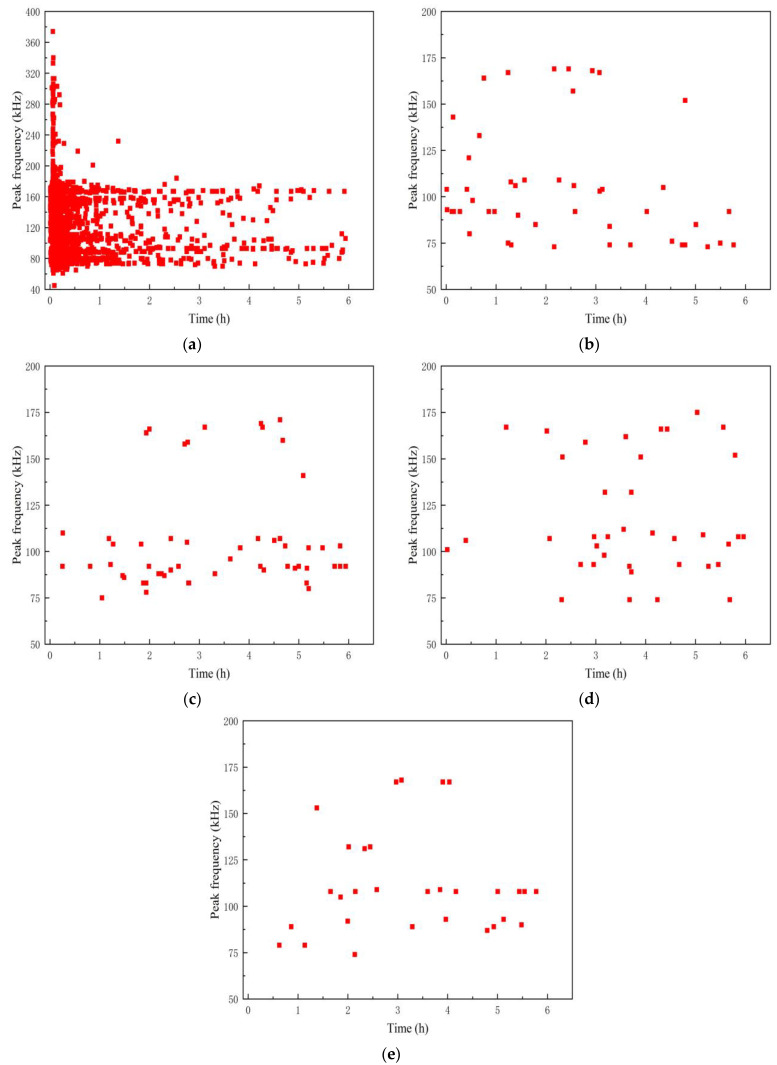
Time–Peak frequency distribution of AE signal in specimen A1. (**a**) Phase 1; (**b**) Phase 2; (**c**) Phase 3; (**d**) Phase 4; (**e**) Phase 5.

As shown in Figure 8, the time–peak frequency relationship of the signal reveals that the peak frequency of the A1 specimen signal is distributed within the range of 70–180 kHz, primarily concentrated between 70–120 kHz and 140–180 kHz, with most signals falling within the 70–120 kHz range. Additionally, the peak frequency of the damage signal during the stress loading period is also distributed in the 200–380 kHz range. Previous studies have shown that fiber fracture signals typically have very short durations and high energies. Combined with the damage sequence of the test piece in the process of stress loading and microscopic observation, this part of the signal corresponds to the fiber fracture damage of the material.

**Figure 9:**
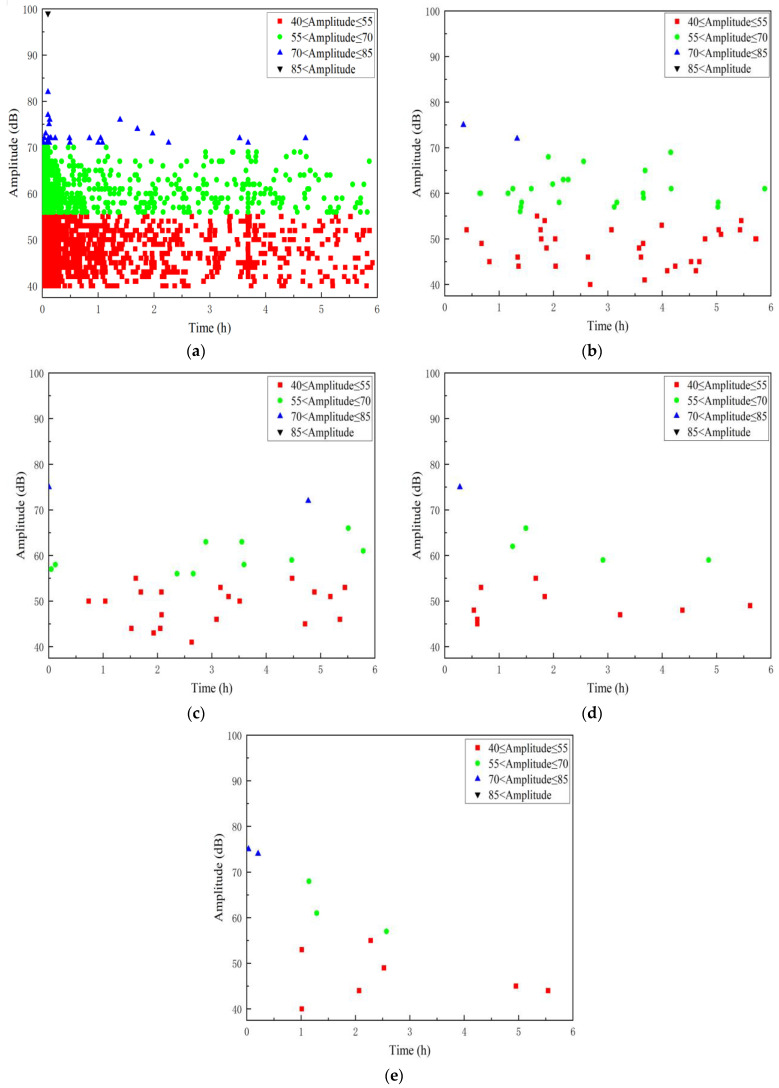
Time–Amplitude distribution of the AE signal in the A2 specimen. (**a**) Phase 1; (**b**) Phase 2; (**c**) Phase 3; (**d**) Phase 4; (**e**) Phase 5.

The A2 specimen is the pipe specimen treated with a 5 mm deep pre-damage. The bending section under pressure has reduced matrix resin and glass fiber content. Stress primarily induces delamination as the main damage pattern. The amplitude ranges mainly between 40 and 70 dB. Compared with the A1 specimen, signals above 70 dB are less frequent. From stage 2 to stage 5, signal amplitudes are predominantly distributed between 40 and 75 dB. Although the aging signal is reduced compared with the A1 specimen, the overall amplitude remains higher. The specific amplitude distribution is depicted in Figure 9.

**Figure 10:**
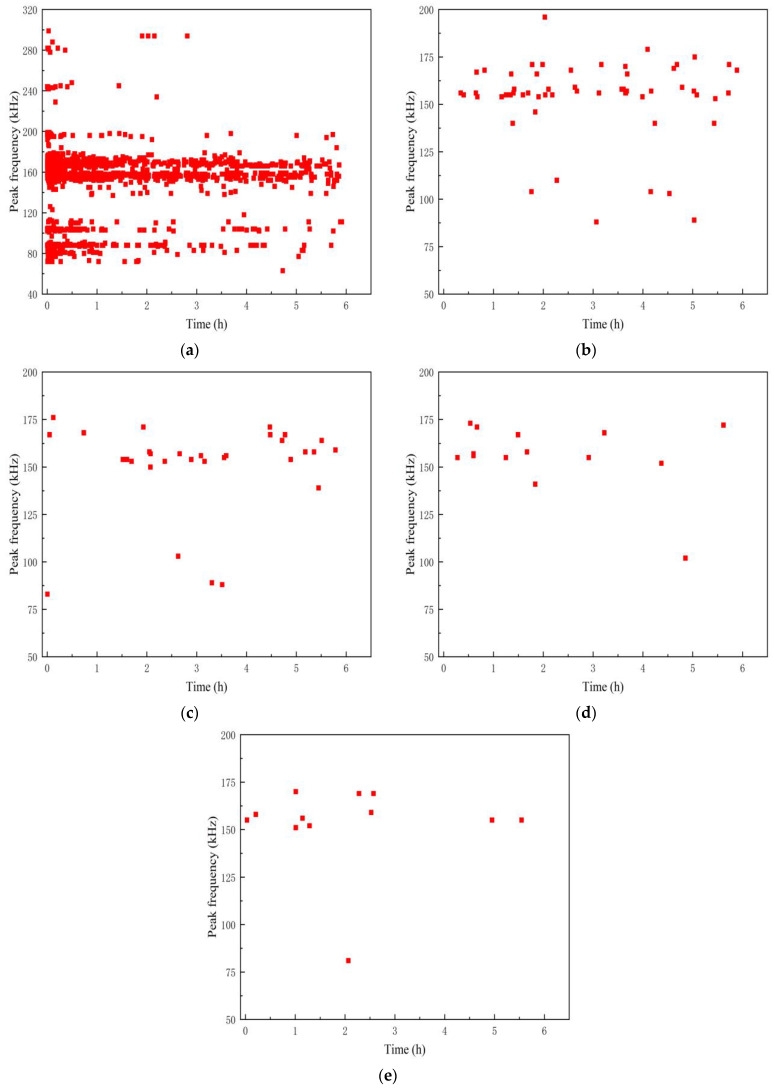
Time–Peak frequency distribution of the AE signal in the A2 specimen. (**a**) Phase 1; (**b**) Phase 2; (**c**) Phase 3; (**d**) Phase 4; (**e**) Phase 5.

As shown in Figure 10, the time–peak frequency distribution characteristics of the aging signal of the A2 specimen are pronounced. Initially, during Phase 1, the peak frequency ranges predominantly between 75~120 kHz and 140~200 kHz, with a notable concentration in the 140~200 kHz range. Subsequently, from the second stage to the fifth stage, frequencies above 150 kHz prevail, although the number of hits diminishes significantly. This suggests that the acidic solution with pH = 5.0 enhances debonding and delamination damage in GFRP pipes to a certain extent, whereas the aging effects on the A1 specimen, characterized by matrix crack damage, are more pronounced.

### 3.3. Cluster Analysis

From the time–amplitude and time–peak frequency analyses of the specimens mentioned earlier, it is evident that the acidic solution exerts varying effects on the aging of GFRP materials under different damage conditions. To further verify the damage types of the A1 and A2 specimens after stress loading, the AE signals from the specimens within the first 10 min of loading were intercepted and analyzed using the K-means clustering analysis.

Through the correlation analysis of the data, the parameters of amplitude, peak frequency, energy, count, and duration of the GFRP pipe specimens were selected. To reduce the complexity of the calculations, a principal component analysis (PCA) was employed to reduce the dimensionality of these high-dimensional characteristic parameters. PCA is a powerful algorithm for data dimension reduction, converting a group of correlated data into several linearly independent variables through orthogonal transformation. These linearly independent variables are known as principal components [26]. Using PCA, two principal components, F1 and F2, were obtained for both specimens. The sampling suitability of the KMO (Kaiser–Meyer–Olkin) measure is greater than 0.6, with values of 0.742, 0.796, 0.716, and 0.774, respectively. The cumulative contribution rate is above 80%, indicating that the data are suitable for principal component analysis [27].

**Figure 11:**
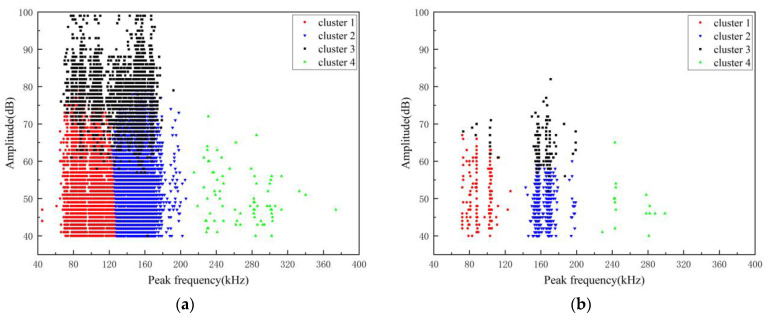
Cluster result. (**a**) A1 specimen; (**b**) A2 specimen.

The damage signals of each specimen can be categorized into four types using the principal components that yield the best effect. These types are named type 1, type 2, type 3, and type 4. Based on the characteristics of these damage signals, the damage evolution process of specimens under stress loading, and microscopic morphology observations, it is found that the four signal types correspond to the matrix damage, debonding, delamination, and fiber fracture of GFRP materials. The clustering effect is displayed in two dimensions, and the damage signals are classified on the peak frequency–amplitude scale, as shown in Figure 11. The number of AE hits on the 2 mm deep V-shaped pre-damaged specimen (A1 specimen) within 10 min is significantly greater than that on the 5 mm deep V-shaped pre-damaged specimen (A2 specimen).

As the loading stress gradually increases, the specimen initially exhibits bending deformation. Subsequently, the stress concentration at the V-shaped defect leads to matrix damage and partial fiber fracture. Delamination damage then begins to occur at the layer where the tip of the V-shaped defect is located, extending to one or both sides. The hits reflect the intensity of the acoustic emission activity of the tested material to a certain extent, i.e., the damage degree of the GFRP pipe test specimens. In an acquisition channel, it is generally considered that the hits are the same as the AE event count, and one hit corresponds to one AE event. Based on Table 4, it is evident that the damage in the A1 specimen is primarily characterized by matrix cracking and debonding. In contrast, in the A2 specimen, matrix damage is no longer the predominant type, and the proportion of delamination damage has significantly increased.

**Figure 12:**
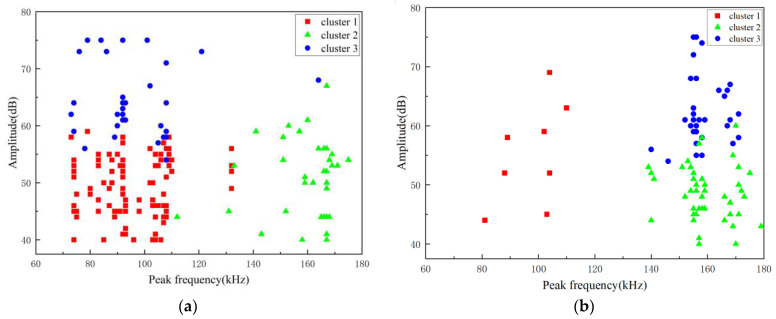
Clustering results of aging signals. (**a**) A1 specimen; (**b**) A2 specimen.

Similarly, a cluster analysis was conducted on the aging signal data of two GFRP materials from Phase 2 to Phase 5 to study the damage types and aging signal characteristics promoted by the acidic aging solution. A K-means clustering analysis was performed on the aging signals of the A1 and A2 specimens by selecting amplitude, peak frequency, duration, and center frequency as parameters. It was found that the clustering effect was optimal when the signals were divided into three types. The clustering results are shown in Figure 12.

The amplitude of the aging signal is concentrated between 40 and 80 dB, while the peak frequency values are concentrated between 70 and 180 kHz. Specifically, Class 1 signals are low-frequency, low-amplitude signals with peak frequencies ranging between 70 and 110 kHz, corresponding to matrix crack damage. Class 2 signals are intermediate-frequency, low-amplitude signals with peak frequencies ranging between 140 and 180 kHz, corresponding to fiber/matrix debonding damage. Class 3 signals are medium-to-low-frequency, high-amplitude signals with peak frequencies ranging between 70 and 170 kHz, corresponding to delamination damage.

The cluster analysis reveals distinct damage characteristics between the A1 and A2 specimens during the stress loading stage. For the A1 specimen, the damage is primarily characterized by matrix cracking and delamination. At this damage level, the aging signals are mainly due to matrix aging, with additional contributions from debonding and delamination. Conversely, for the A2 specimen, the damage is predominantly due to debonding and delamination. At this more severe damage level, the aging signals are primarily due to delamination, with debonding acting as a secondary contributor and matrix aging being the least significant.

As shown in Table 5, the number of fiber/matrix debonding signals is greater for the pre-damaged specimens with a 5 mm deep V-shaped defect compared with those with a 2 mm deep V-shaped defect. This can be attributed to two main factors. First, the larger pretreatment depth of the 5 mm groove increases the contact surface area between the aging solution and the interior of the material. Second, due to the severe delamination damage, the aging solution penetrates and diffuses into the material through multiple channels, such as the delaminated areas, which reduces the mechanical properties and accelerates the aging process.

### 3.4. Least Squares Support Vector Machine Algorithm for Classified Prediction

The least squares support vector machine (LSSVM) algorithm, proposed by Su, is an enhancement of the traditional support vector machine (SVM) algorithm. While the SVM algorithm yields good prediction results in classification modeling, it suffers from complex models, large computational requirements, and low efficiency in solving convex quadratic programming problems [28]. LSSVM retains the powerful learning and generalization capabilities of the SVM algorithm but improves the efficiency by converting the convex quadratic programming problem into a linear solution problem. This is achieved by defining the Lagrange function and employing a least squares algorithm, thereby reducing model complexity and calculation time [29].

Assuming the data set $S=\left\{\left(x_{i},y_{i}\right)|i=1,2,3,\ldots ,n\right\}$, where $y_{i}$ represents the output data, the calculation process for the high-dimensional mapping of feature data is:

(1) $$y_{i}=w^{T}\phi (x_{i})+b+e_{i},i=1,2,3,\ldots n$$

where w is a normal vector, $\phi (x_{i})$ is the nonlinear mapping function, b is the amount of displacement, and $e_{i}$ is relaxation variables.

Equation (1) is the constraint condition under which LSSVM controls all samples and errors. The optimization problem is:

(2) $$\underset{w,b,e}{\min}J(w,e)=\frac{1}{2}w^{T}w+\frac{1}{2}\gamma^{\prime }\sum_{i=1}^{n}e_{i}^{2}$$

where $\gamma^{\prime }$ is a regularization parameter and J(w,e) is an optimization function.

Therefore, for the above optimization problem, the Lagrange function set by LSSVM is:

(3) $$\begin{array}{l} L(\omega ,b,e,\alpha )=J(w,e)-\sum_{i=1}^{n}\alpha_{i}\left\{w^{T}\phi (x_{i})+b+e_{i}-y_{i}\right\}\\ =\frac{1}{2}w^{T}w+\frac{1}{2}\gamma^{\prime }\sum_{i=1}^{n}e_{i}^{2}-\sum_{i=1}^{n}\alpha_{i}\left\{w^{T}\phi (x_{i})+b+e_{i}-y_{i}\right\} \end{array}$$

where $\alpha_{i}$ is the Lagrange multiplier. Solve partial derivative for ω,b,e,α, respectively, and eliminate ω,e to obtain a linear equation set:

(4) $$\left[\begin{array}{cc} 0 & D^{T}\\ D & Q_{i,j}+\frac{1}{\gamma^{\prime }}I \end{array}\right]\left[\begin{array}{l} b\\ a \end{array}\right]=\left[\begin{array}{l} 0\\ y \end{array}\right]$$

where $y=\left[y_{1},y_{2},y_{3}\ldots y_{n}\right]$, $\alpha ={\left[\alpha_{1},\alpha_{2},\alpha_{3,}\ldots \alpha_{n,}\right]}^{T}$, $D={\left[1,1,1,\ldots 1\right]}_{n}^{T}$. According to the Mercer condition,

(5) $$Q_{i,j}=y_{i}y_{j}\phi {\left(x_{i}\right)}^{T}\phi (x_{i})=y_{i}y_{j}K(x_{i},x_{j})$$

It can be obtained through Formulas (3) and (5),

(6) $$y(x)=\sum_{i=1}^{n}\alpha_{i}K(x_{i},x_{j})+b$$

Based on the above analysis, the LSSVM model calculation is straightforward. In this paper, the Gaussian kernel function is selected for model calculation.

The LSSVM model requires the determination of the kernel function and the normalized parameter *C*. Both the normalized parameter and the penalty factor serve similar roles, balancing the error size of training results against the complexity of the model. The kernel function choice influences the number of support vectors; an excessively large value can lead to model simplicity, resulting in underfitting and reduced prediction accuracy. Before modeling, it is crucial to select appropriate kernel functions and normalized parameter *C* values, as these parameters significantly impact the accuracy of model training. Intelligent algorithms are increasingly employed to find optimal parameters. In this study, a swarm intelligence optimization technique is proposed, which is called the sparrow search algorithm (SSA). The SSA is utilized to identify optimal penalty factors and kernel function parameters. Introduced by Xue in 2020 [30], the SSA is a group-based intelligent optimization algorithm known for its rapid convergence and robust local search capabilities. The SSA has found applications in various domains, including logistics site selection, rolling bearing fault diagnosis, and agricultural network detection.

In the sparrow search algorithm, the population can be represented by *X*, which represents n sparrows in the d-dimension space.

(7) $$X=\left[\begin{array}{cccc} x_{1}^{1} & x_{1}^{2} & \ldots & x_{1}^{d}\\ x_{2}^{1} & x_{2}^{2} & \ldots & x_{2}^{d}\\ \ldots & \ldots & \ldots & \ldots \\ x_{n}^{1} & x_{n}^{2} & \ldots & x_{n}^{d} \end{array}\right]$$

Population fitness can be expressed as *F*, which is the individual fitness value of sparrows:

(8) $$F=\left[\begin{array}{l} f([\begin{array}{cccc} x_{1}^{1} & x_{1}^{2} & \ldots & x_{1}^{d} \end{array}])\\ f(\begin{array}{cccc} {}[x_{2}^{1} & x_{2}^{2} & \ldots & x_{2}^{d}] \end{array})\\ \begin{array}{c} \;\;\;\;\vdots \end{array}\\ f(\begin{array}{cccc} {}[x_{n}^{1} & x_{n}^{2} & \ldots & x_{n}^{d} \end{array}]) \end{array}\right]$$

The finder usually finds the food with the highest fitness value, accounting for about 20% of the population. Its location iterative formula is shown in (9). When $R_{2}<ST$, it means that no danger is detected within the population, and individuals within the population can update their positions; when $R_{2}\geq ST$, it indicates the presence of danger such as predators within the population, prompting the birds to migrate for self-preservation.

(9) $$X_{i,j}^{t+1}=\left\{\begin{array}{l} X_{i,j}^{t}\cdot \exp\left(1\frac{i}{\alpha \cdot T}\right)\;\;\;\;R_{2}<ST\\ X_{i,j}^{t}+Q\cdot L\;\;\;\;\;\;\;R_{2}\geq ST \end{array}\right.$$

where t is iteration, $X_{i,j}^{t}$ is the position of the *i*th individual of the *j*th dimension at iteration t, α is a constant between $\left(0,1\right]$, $R_{2}$ is the pre-alarm value in the range $\left(0,1\right]$, T is the maximum iteration times, ST is the safety value in the range $\left(0.5,1\right]$, and L is 1 × d matrix with all elements equal to 1.

The sparrow that is assigned the role of the follower within the population can continually monitor the location of the finder. When the finder discovers better food, the follower promptly seizes the opportunity. The iterative formula governing the follower’s position is as follows, highlighting that if the fitness of the *i*th follower is low, it must relocate to another location:

(10) $$X_{i,j}^{t+1}=\left\{\begin{array}{l} Q\cdot \exp\left(\frac{X_{worst}-X_{i,j}^{t}}{i^{2}}\right),\;i>\frac{n}{2}\\ X_{p}^{t+1}+|X_{i,j}^{t}-X_{p}^{t+1}|A^{+}\cdot L \end{array}\right.$$

where $X_{worst}$ is the worst case global location, $X_{p}^{t+1}$ is the best position for the finder at iteration *t* + 1, and A is a 1 × d matrix with random elements of 1 or −1, *A*^+^ = *A*^T^(*AA*^T^)^−1^.

The sparrow designated as the scout within the population, responsible for detecting danger and issuing warnings, operates according to the following location iterative formula:

(11) $$X_{i,j}^{t+1}=\left\{\begin{array}{l} X_{best}^{t}+\beta \cdot |X_{i,j}^{t}-X_{best}^{t}|,\;f_{i}>f_{g}\\ X_{i,j}^{t}+K\cdot \left(\frac{\left|X_{i,j}^{t}-X_{worst}^{t}\right|}{(f_{i}-f_{w})+\varepsilon }\right),\;f_{i}=f_{g} \end{array}\right.$$

where $X_{best}$ is the global optimum location, β,K is the step control parameter, ε is a constant to guarantee that the molecule is not 0, $f_{i}$ is the fitness value of the current individual, and $f_{g},f_{w}$ is the global optimum and worst fitness value.

The detailed process of optimizing the LSSVM algorithm using the SSA method includes the following steps: (1) establishing tagged stress damage-oriented data sample sets and aging-oriented data sample sets; (2) normalizing the sample data and selecting a training set and a test set; (3) determining penalty factors and kernel function parameters of the SSA optimization algorithm using the training samples; and (4) evaluating the accuracy of the LSSVM algorithm modeling and model recognition.

**Figure 13:**
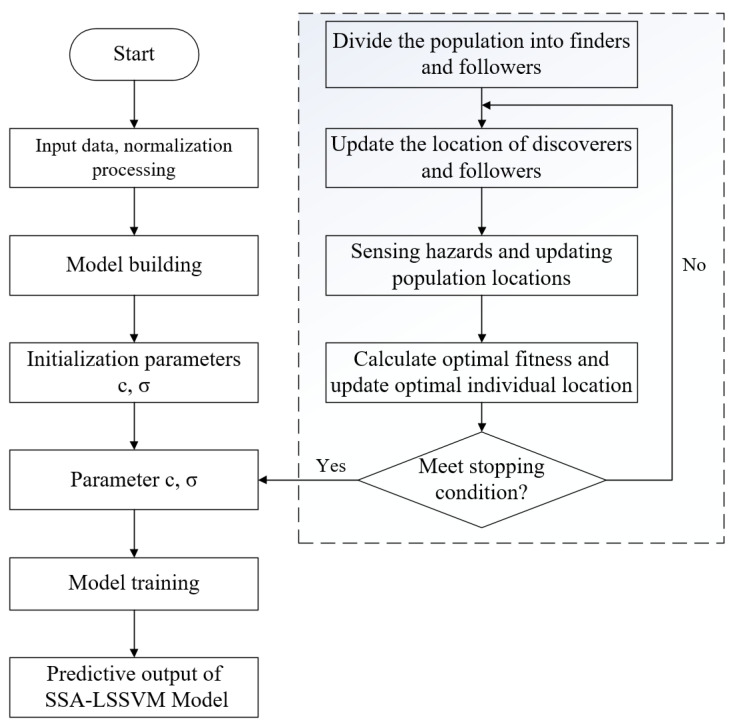
Process of the SSA-LSSVM algorithm.

To summarize, this paper adopts the SSA-LSSVM algorithm for modeling and predicting data classification. The specific algorithm flow is illustrated in Figure 13.

Taking the A1 specimen as an example, the AE signal is segmented into two parts: stress damage and aging. A prediction model was established using the LSSVM approach. For the stress damage part: Training set: 100 signals of matrix cracking, debonding, and delamination after clustering, along with 50 signals of fiber fracture. Test set: 30 signals of matrix cracking, debonding, and delamination, along with 20 signals of fiber fracture after clustering. For the aging failure part: Training set: 60 matrix crack signals after clustering, 20 debonding signals, and 20 delamination signals. Test set: 30 matrix crack signals, 10 debonding signals, and 10 delamination signals after clustering. The composition and quantity of acoustic emission data samples used for model training and testing are detailed in Table 6.

**Figure 14:**
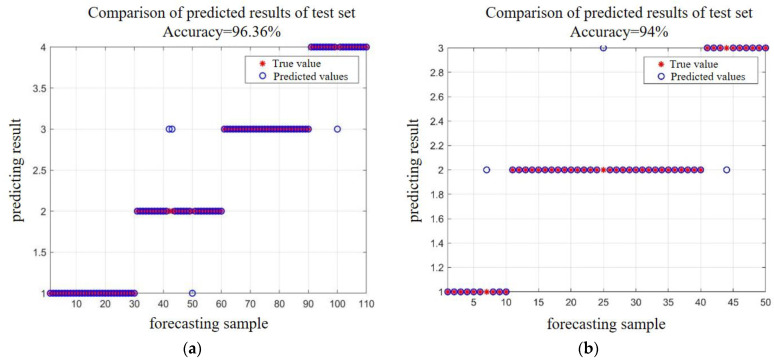
Prediction results. (**a**) Stress damage stage; (**b**) aging stage.

Furthermore, characteristic parameters such as amplitude, peak frequency, energy, count, duration, and center frequency were selected as input features for the LSSVM. SVM characteristic parameters were initialized, and both damage signal samples and aging signal samples were trained and classified using these parameters. The prediction results are illustrated in Figure 14.

## 4. Conclusions

This paper investigates the stress aging behavior of GFRP pipe specimens exhibiting matrix cracking and delamination damage in an acidic solution with pH = 5.0. The experiments are divided into two stages: the stress damage stage and the aging stage. Different types of damage in GFRP materials are identified through a cluster analysis, and the aging promotion effect of the acidic solution on various specimens is analyzed. The SSA-LSSVM algorithm is utilized for the pattern recognition of damage and aging signals. The main conclusions are as follows:

(1) The acid aging solution accelerates matrix damage, fiber/matrix debonding, and delamination of GFRP pipes in the short term; however, its overall aging effect is mild. This underscores that GFRP pipes, as alternatives to steel pipes in oil and gas fields, exhibit robust corrosion resistance and significant advantages.
(2) The matrix aging signal in GFRP manifests as a low-frequency, low-amplitude signal peaking between 70 and 110 kHz. In contrast, the fiber/matrix debonding signal appears as a high-frequency, medium-to-low-amplitude signal with a peak frequency of 140 to 180 kHz. Signals indicative of layered damage show overlapping frequencies mainly ranging from 70 to 170 kHz, characterized by high amplitudes.
(3) This paper employs the SSA-LSSVM algorithm to develop a predictive model with excellent identification capabilities. The model achieves high identification accuracies of 96% for the stress damage stage and 94% for the aging stage.
(4) In the future, the field gathering and transmission medium can be used as the aging solution to monitor the specimens for a longer time and further improve the pipeline aging database for the identification of damage degree and damage type of GFRP pipes.

## Figures and Tables

**Table 1:** Parameters of the GFRP pipe.

Angle	Thickness		
Winding angle	Inner liner	Inner winding layer	Outer winding layer
54.75°	3 mm	3 mm	1.5 mm

**Table 2:** Composition of aging solution.

Ion	Cl^−^	HCO_3_^−^	Ca^2+^	Mg^2+^
concentration (g/L)	15	0.8	0.8	0.8

**Table 3:** Design of the specimen.

Preprocessed Specimens	Specimen Number	Degree of Damage
2 mm	A1	Matrix crack
5 mm	A2	Obvious delamination

**Table 4:** Hits during the damage process of various specimens.

Damage Type	Hits			
	Matrix Cracking	Debonding	Delamination	Fiber Fracture
A1	9676	21,621	2863	77
A2	162	457	170	17

**Table 5:** Hits during the aging process of various specimens.

Damage Type	Hits		
	Matrix Crack	Debonding	Delamination
A1	99	37	34
A2	8	47	34

**Table 6:** Model components.

Model	Experimental Stage	Sample Number of Training Set	Sample Number of the Testing Set
Model 1	Stress damage stage	350	110
Model 2	Aging stage	100	50

## Data Availability

The original contributions presented in the study are included in the article; further inquiries can be directed to the corresponding authors.
